# Histological Assessment of the Anti-Inflammatory Effectiveness of *Peperomia pellucida* Extract Administered to the Gingival Sulcus in Rats Induced with Periodontitis

**DOI:** 10.1055/s-0045-1802950

**Published:** 2025-03-12

**Authors:** Dewi Lidya Ichwana Nasution, Sri Tjahajawati, Ratna Indriyanti, Amaliya Amaliya, Widya Irsyad, Indah Puti Sabirin

**Affiliations:** 1 Department of Dentistry, Faculty of Dentistry, Padjadjaran University, Jawa Barat, Indonesia; 2 Department of Periodontic, Faculty of Dentistry, Jenderal Achmad Yani University, Jawa Barat, Indonesia; 3 Departement of Oral Biology, Faculty of Dentistry, Padjadjaran University, Jawa Barat, Indonesia; 4 Departement of Pediatric Dentistry, Faculty of Dentistry, Padjadjaran University, Jawa Barat, Indonesia; 5 Department of Periodontic, Faculty of Dentistry, Padjadjaran University, Jawa Barat, Indonesia; 6 Departement of Orthodontic, Faculty of Dentistry, Jenderal Achmad Yani University, Jawa Barat, Indonesia; 7 Departement of Oral Biology, Faculty of Dentistry, Jenderal Achmad Yani University, Jawa Barat, Indonesia

**Keywords:** *Peperomia pellucida*, periodontitis, anti-inflammatory, antimicrobial activity, Wistar rats

## Abstract

**Objective:**

This study aims to assess the impact of *Peperomia pellucida* extract on periodontitis in rats, using the Papillary Bleeding Index (PBI), gingival index (GI), and histological evaluation of key inflammatory cells such as osteoclasts, osteoblasts, polymorphonuclear neutrophils (PMNs), macrophages, and fibroblasts to explore its potential in reducing inflammation and preserving periodontal tissue.

**Materials and Methods:**

The extract was prepared using the reflux method with 96% ethanol as a solvent, followed by phytochemical screening and antibacterial testing via the disk diffusion method. This *in vivo* study utilized a posttest control group experiment with 24 Wistar rats, divided into four groups: nonperiodontitis, no-treatment, chlorhexidine-treated (CHX), and extract-treated groups, with the latter three groups induced with periodontitis. Induction was performed using a 0.3-mm ligature wire and plaque from periodontitis patients, along with nicotine administration (0.001 mg/L) for 7 days. The extract group received a topical application of 2.5 µL of *P. pellucida* leaf extract, while the CHX group was administered 0.05 mL of CHX daily for 1 week. Observations of GI and PBI were made on days 0, 3, 5, and 7. Histological changes were assessed on day 7 by evaluating the cell counts of osteoclasts, osteoblasts, fibroblasts, macrophages, and PMNs.

**Statistical Analysis:**

Data were analyzed using one-way analysis of variance and Kruskal–Wallis with Mann–Whitney post hoc tests for pairwise comparisons.

**Results:**

Phytochemical analysis confirmed the presence of alkaloids, polyphenols, tannins, flavonoids, quinones, monoterpenoids, and sesquiterpenoids in *P. pellucida* extract. The extract demonstrated antibacterial activity against *Porphyromonas gingivalis* , a key pathogen in periodontitis. Clinical and histological assessments showed significant improvements in the extract-treated group, with outcomes comparable to the CHX-treated group after 7 days.

**Conclusion:**

Based on these findings, *P. pellucida* (L.) Kunth extract contains phytochemicals and exhibits antibacterial and anti-inflammatory properties, as demonstrated by clinical and histological parameters in rats induced with periodontitis.

## Introduction

Periodontitis is a chronic multifactorial inflammatory disease associated with the accumulation of dental plaque and characterized by the progressive destruction of periodontal tissues, including the periodontal ligament, cementum, and alveolar bone. Periodontitis involves a complex dynamic interaction between specific bacterial pathogens and a damaging immune response. 1 2 3 4 Patients with advanced and difficult-to-control periodontitis, with a gingival attachment loss greater than 6 mm, require additional treatment besides scaling and root planing (SRP). 5 6 Surgical treatment with periodontal flap after SRP provides better results in terms of reduced probing depth and increased clinical attachment. Periodontal surgical treatment requires expertise, professional experience, adequate tools, and tends to cause greater anxiety in patients. Additionally, certain systemic diseases must be considered before deciding on surgical therapy. 7 8 Many studies have discussed adjunctive antibiotic therapy for periodontitis. This therapy is much more affordable, easier, and relatively safe for short-term use, but it has some drawbacks that need to be considered, particularly: side effects with long-term use, the potential for allergic reactions, bacterial resistance, drug toxicity, and the need for higher doses to achieve the required concentration of gingival crevicular fluid at the target site. 9 10 11 12 13

Herbal medicines and formulations consisting of plant elements have been recognized for their therapeutic benefits. The advantages of herbal products compared with chemical drugs include having natural activity, a higher safety margin, and lower cost. 14 Many studies have proven that the use of herbal medicines as an adjunct therapy to SRP in the treatment of periodontitis has a better reparative effect on periodontal tissues compared with SRP alone. This has been evaluated through periodontal parameters such as reductions in the gingival index (GI), plaque index, bleeding index, and periodontal probing depth, along with a significant increase in the level of tissue attachment. 9 15 16 17 18 19 20 21 The addition of herbal substances in periodontal treatment, whether locally or systemically, has shown a significant reduction in the enzymatic activity of microorganisms, decreased the number of pathogenic bacteria in the oral cavity, and reduced inflammatory markers. 9 22

*Peperomia pellucida* , commonly known as pepper elder, is a herbal plant with broad pharmacological potential. Previous studies have demonstrated its antibacterial activity, where extracts of *P. pellucida* effectively inhibit the growth of *Propionibacterium acnes* . 23 24 Additionally, it exhibits significant antioxidant activity and contains high levels of bioactive compounds. 25 26 The plant also shows promise as an antidiabetic agent through its ability to inhibit the enzyme α-glucosidase and has been traditionally utilized for antihypertensive purposes. 27 28 29 Furthermore, several studies have reported the cytotoxic activity of *P. pellucida* extracts against certain cancer cell lines, positioning it as a potential candidate for the development of anticancer therapies. 25 These findings highlight the remarkable potential of *P. pellucida* as a natural bioactive compound, supporting its application in innovative therapeutic developments across various fields of health and medicine. 25 26 27 28 29 30 31 32 However, empirical evidence on the effectiveness of *P. pellucida* extract in treating periodontitis remains limited, especially regarding its effects on specific cells such as osteoclasts, osteoblasts, fibroblasts, macrophages, and polymorphonuclear neutrophils (PMNs). Osteoclasts are responsible for increased bone resorption under inflammatory conditions, while osteoblasts play a critical role in forming new bone, essential for tissue regeneration. Fibroblasts contribute to periodontal tissue integrity by producing collagen and extracellular matrix, and macrophages and PMNs are immune cells that participate in the inflammatory response to bacterial infection. 28 29

Various models of periodontitis in laboratory animals have been developed to study the pathogenesis of periodontitis and the effectiveness of new treatment methods across different animal species. Small rodent models are the most commonly used due to their ease of handling and the ability to reproduce inflammatory processes in periodontal tissue. The most frequently employed method is the ligature-induced periodontitis model, where a ligature placed around the cervical area of the tooth causes bacterial colonization and the development of inflammation. Moiseev et al developed a new method to accelerate periodontitis modeling in mice, using a combination of local factors designed to disrupt the functional integrity of the periodontal tissue within a short period (7 days). 33 A period of 7 days can initiate an acute inflammatory response in gingival tissue, marked by an influx of inflammatory cells such as macrophages and PMNs, and may begin to reveal initial shifts in the balance between osteoclasts and osteoblasts. This duration allows for an observable increase in osteoclast activity due to inflammation, potentially leading to early-stage bone resorption. However, significant changes in osteoblast numbers, essential for new bone formation, generally require a longer time frame to fully capture both the destructive and regenerative processes in periodontal tissues. Thus, a 7-day period may adequately reflect initial gingival inflammation and osteoclastic activity, though it may not fully represent the advanced destructive or regenerative alterations in periodontal structures. 34 35

This study aims to evaluate the effectiveness of *P. pellucida* extract in a rat model of induced periodontitis, with periodontitis induction for 7 days followed by extract administration for an additional 7 days. At the study's conclusion, the periodontal tissues of the rats will be histologically analyzed to quantify osteoclasts, osteoblasts, fibroblasts, macrophages, and PMNs. This evaluation is expected to provide empirical insights into the potential of *P. pellucida* extract to reduce inflammatory and resorptive cell activity, thereby protecting periodontal tissue from further degradation.

## Materials and Methods

This research was an experimental laboratory study using a post-test control group design with four groups of *Rattus norvegicus* Wistar rats. The study tested the effectiveness of *P. pellucida* (L.) Kunth leaf extract as an adjunct treatment for periodontitis induction. It was conducted at the Pharmacy Laboratory and Animal Laboratory of the Faculty of Medicine, Jenderal Achmad Yani University, West Java, Indonesia.

The inclusion criteria for *P. pellucida* (L.) Kunth leaves required them to be mature, dark green, and intact (free from malformations, insect damage, or disease) and harvested from the upper parts of the plant. The inclusion criteria for the *Rattus norvegicus* Wistar rats specified that they had to be male, aged 2 to 3 months, with an average weight of 200 to 250 g, and in good health, indicated by active movement, no wounds or body defects, and a good response to stimuli. Rats that died during the study or had their ligature wires detached from the interdental space before the 7-day period of periodontitis induction were excluded from the study. 2 32 33

The sample size was determined using the Federer formula, which indicated a minimum of six rats per group, resulting in a total of 24 rats. The groups were as follows: (1) healthy rats without induced periodontitis (no periodontitis group), (2) rats with induced periodontitis without treatment (no treatment group), (3) rats with induced periodontitis treated with 0.2% chlorhexidine gluconate (CHX-treated group), and (4) rats with induced periodontitis treated with *P. pellucida* extract (extract-treated group).

### Preparation *P. pellucida* Leaf Extract

Preparation of *P. pellucida* leaf extract was performed using the reflux method. The reflux method is an extraction technique that uses a solvent at its boiling point for a specific duration, with a relatively constant amount of solvent and a reflux condenser. In the context of colorant extraction, this method utilizes the solvent as the extraction medium, with water being one of the effective and commonly used polar solvents. 36 The basic principle of the reflux method involves using a solvent that is then condensed to return it to a liquid state and back into the reaction vessel. The solvent remains present throughout the reaction, allowing the process to proceed effectively. 36 37

A total of 1,000 g of *P. pellucida* leaves were accurately weighed, thoroughly washed, and oven-dried. The dried leaves were then ground into a fine powder and transferred to a round-bottom flask. The powdered leaves were macerated in 96% ethanol, and the flask was equipped with a reflux condenser and heated for 1 hour. Following the extraction, the mixture was filtered through filter paper, and the solvent in the filtrate was removed using a rotary evaporator, yielding the crude *P. pellucida* leaf extract. This extract was subsequently concentrated further in a water bath. The concentrated extract was then diluted with dimethyl sulfoxide to obtain a 50% solution. 36

### Phytochemistry of *P. pellucida* Leaf Extract

Phytochemical screening of *P. pellucida* (L.) Kunth extracts was conducted to identify the presence of alkaloids, polyphenols, tannins, flavonoids, quinones, saponin, monoterpenoids, sesquiterpenoids, steroids, and triterpenoids. 38

*Test for alkaloids* : The extract was basified with ammonia, followed by the addition of chloroform, and then vigorously shaken. The chloroform layer was pipetted out and treated with 2 N hydrochloric acid. The mixture was shaken until two layers formed. The acid layer was pipetted out and divided into three portions:

- Portion 1: Mayer's reagent was added. The formation of a white precipitate or turbidity indicated the possible presence of alkaloids.
- Portion 2: Dragendorff's reagent was added. The formation of an orange-yellow to brick-red precipitate suggested the possible presence of alkaloids.
- Portion 3: This portion was used as a blank.

*Test for polyphenols* : The extract was placed in a test tube, distilled water was added, and the mixture was heated over a water bath before filtration. Ferric chloride solution was then added to the filtrate. The development of a green-blue to black color indicated the presence of phenolic compounds.

*Test for tannins* : The extract was placed in a test tube, distilled water was added, and the mixture was heated over a water bath, followed by filtration. The filtrate was divided into two portions:

- Portion 1: Addition of a few drops of 1% gelatin solution led to the formation of a white precipitate, indicating the presence of tannins.
- Portion 2: Addition of Steasny's reagent produced a pink precipitate, confirming the presence of tannins.

*Test for flavonoids* : The extract was placed in a test tube with distilled water, magnesium powder, and 2 N hydrochloric acid. The mixture was heated over a water bath and then filtered. Amyl alcohol was added to the filtrate in a new test tube, followed by vigorous shaking. The development of a yellow to red color in the amyl alcohol layer indicated the presence of flavonoids.

*Test for quinones* : The extract was placed in a test tube, distilled water was added, and the mixture was heated over a water bath and then filtered. A 5% pottasium hydroxide (KOH) solution was added to the filtrate. The development of a yellow color indicated the presence of quinone compounds.

*Test for saponins* : The extract was placed in a test tube, distilled water was added, and the mixture was heated over a water bath and then filtered. The filtrate was shaken and left to stand for 5 minutes. The formation of foam, which persisted after the addition of dilute HCl, indicated the presence of saponin compounds.

*Test for monoterpenoids and sesquiterpenoids* : The extract was ground with ether and filtered. The filtrate was placed in an evaporating dish and allowed to evaporate until dry. A 10% vanillin solution in concentrated sulfuric acid was added to the residue. The appearance of colors indicated the presence of monoterpenoid–sesquiterpenoid compounds.

*Test for steroids and triterpenoids* : The extract was ground with ether and filtered. The filtrate was placed in an evaporating dish and allowed to evaporate until dry. Liebermann–Burchard reagent was added to the residue. The development of a purple color indicated the presence of triterpenoid compounds, while a green-blue color indicated the presence of steroid compounds.

### Antibacterial Assay

The antibacterial activity against *Porphyromonas gingivalis* was assessed using the disc diffusion method. Bacterial inoculation was performed using a sterile loop in liquid media and incubated anaerobically for 24 hours at 37°C. After standardization with McFarland standard 0.5, bacterial seeding was performed on agar media using the spread plate technique. *P. pellucida* extracts at concentrations of 25, 50, 75, and 100% were applied to sterile disc paper using a sterile micropipette at a volume of 0.01 mL. The discs were then placed on the agar surface and incubated for 48 hours under aerobic conditions at 37°C. Clear zones around the paper discs were observed and measured using a caliper, and the diameters of the inhibition zones were interpreted according to the classification by David and Stoud (2021): > 20 mm, very strong; 10 to 20 mm, strong; 5 to 10 mm, moderate; and < 5 mm, no response. The antibacterial testing was conducted at the Microbiology Laboratory, Faculty of Dental Medicine, Airlangga University, Surabaya, Indonesia. 39 40

### Preparation of Animal Research

The preparation for the study involved setting up both the subjects and the materials required, including equipment and supplies. The study was approved by the local Ethics Committee of Faculty of Medicine, Jenderal Achmad Yani University, West Java, Indonesia No: 032/UH4.11/2023. The research object was *P. pellucida* leaf extract, and the research subjects were *Rattus norvegicus Wistar* rats obtained from the Laboratory of the Faculty of Medicine, Jenderal Achmad Yani University, meeting the inclusion criteria. The rats were acclimated for 1 week prior to the experimental period in the animal laboratory, maintained at a temperature of 22 to 23°C with a 12-hour light/dark cycle. The rats were provided with *ad libitum* access to water and commercial rodent pellets. All animals were treated in accordance with the guidelines from the National Academy of Sciences for the care and use of laboratory animals. 2 33 41 42 The animals are randomly divided into four groups: no periodontitis (healthy group), induced periodontitis without treatment (negative control), induced periodontitis treated with CHX (positive control), and induced periodontitis treated with *P. pellucida* extract.

### Experimental Induced Periodontitis in Animal Studies and Administration of Extract

Periodontitis induction in animal studies was performed in three groups: the negative control group, the positive control group, and the *P. pellucida* extract group. This study used two dentoalveolar segments per rat, specifically in the interdental cervical areas of the left and right upper incisors of Wistar rats. The selection of these teeth for periodontitis induction was based on their accessibility.

All periodontitis induction procedures were performed under intraperitoneal anesthesia using ketamine (HCl) 40 to 80 mg/kg body weight and xylazine (Xyla) 5 to 10 mg/kg body weight, with anesthesia lasting approximately 1 hour. Before placing the ligature wire, the interdental area of the teeth was exposed using 2.5 mm silk thread (Onemed). A ligature wire (OSU) with a diameter of 0.3 mm was then placed in an eight-shaped pattern around the cervical area of the left and right upper incisors. The ends of the wire were twisted with a needle holder and ligature wire tucker until securely fastened. The cut ends of the wire were bent to prevent injury to the animal's oral mucosa and gingiva.

Dental plaque was collected from a patient with severe chronic periodontitis using a Gracey curette (UNC 15, Osung MND Co., Seoul, Korea), scraped from the crown and cervical areas of the teeth, and stored in a sterile container for no more than 2 hours. The plaque was placed to fill the interdental space up to the ligature wire using a sterile paper point #40, and adherence of the plaque was ensured. Rats with ligature wire and plaque from the periodontitis patient were then given a nicotine solution in physiological saline (0.001 mg/L), injected submucosally into the gingiva in a volume of 0.05 mL. This nicotine solution was administered once daily for 7 days. 33

The ligature wire placed around the cervical area of the rats was removed on the seventh day or when periodontitis was confirmed, as indicated by changes in gingival color and consistency, gingival bleeding upon probing, and increased probing depth of the periodontal pocket. Rats confirmed with periodontitis were then subjected to measurements and evaluations of the Papillary Bleeding Index (PBI) and GI.

Two rats were randomly selected to represent the sample group, anesthetized, and then decapitated to obtain their jaws, which were subsequently fixed in a 70% alcohol solution. Histological examination was performed to assess the number of macrophages, neutrophils, osteoclasts, osteoblasts, and fibroblasts. Experimentally induced periodontitis in rats was confirmed by clear infiltration of inflammatory cells, particularly neutrophils, in the gingival tissue, increased osteoclasts, decreased osteoblasts, and resorption of the alveolar process in hematoxylin and eosin (H&E)-stained sections. 43 44 45

The group without induced periodontitis and the negative control group received no treatment, while the positive control group received a topical application of 0.2% CHX (Minosep) around the periodontitis-induced teeth, administered in a 0.05 mL dose. 9 46 47 48 The extract group received a topical application of 2.5 µL of *P. pellucida* leaf extract around the periodontitis-induced teeth, the dosage of 2.5 µL of *P. pellucida* was established based on prior research, which demonstrated that a 50% concentration showed potent antibacterial effects against *P. gingivalis* and *Aggregatibacter actinomycetemcomitans* , the primary bacteria associated with periodontitis. 32 40 CHX and extract treatments for the positive control and extract groups were given once daily in the afternoon for 1 week.

### Papillary Bleeding Index and Gingival Index Examination

The PBI and GI were assessed on the same day the ligature wire is removed (confirming periodontitis), and on days 3, 5, and 7 afterward. PBI and GI values were compared before and after treatment.

**Fig. 1:**
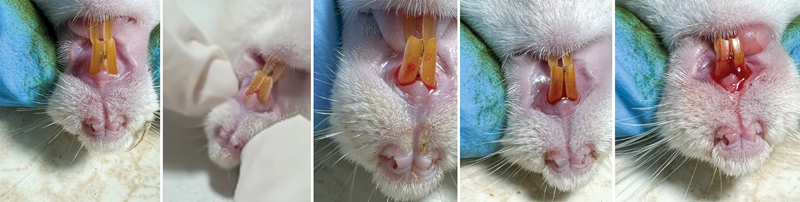
Interpretation of Papillary Bleeding Index results in rats with induced periodontitis.

A visual representation of the PBI is provided using a scale with the following grades ( Fig. 1 ):

- Grade 0: No bleeding
- Grade 1: Bleeding points
- Grade 2: Bleeding lines
- Grade 3: Bleeding triangles
- Grade 4: Diffuse bleeding

**Fig. 2:**
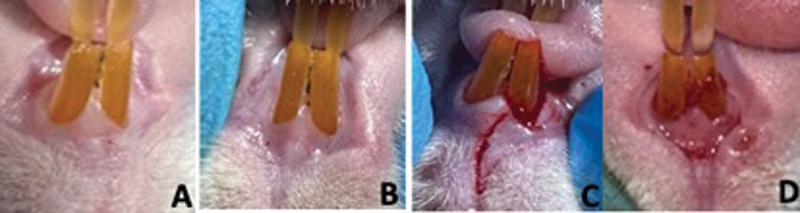
Interpretation of gingival index (GI) results in rats with induced periodontitis. ( **A** ) GI 0, ( **B** ) GI 1, ( **C** ) GI 2, and ( **D** ) GI 3.

The condition of the gingiva is assessed on two upper front teeth in rats with induced periodontitis. The GI is evaluated using the following scores and criteria ( Fig. 2 .):

- 0: No inflammation of the gingiva
- 1: Mild inflammation characterized by slight color change and swelling, with no bleeding upon probing
- 2: Moderate inflammation with redness, swelling, and shininess, with bleeding upon probing
- 3: Severe inflammation with more pronounced redness and swelling, ulceration, and spontaneous bleeding 49

### Histological Assay

On the seventh day, the rats were anesthetized and decapitated with an overdose of ether administered intramuscularly, followed by neck dislocation. All animals were anesthetized with a mixture of Zoletil (Virbac) and Rompun (Bayer) (1 mL/kg) administered intraperitoneally (4:1 v/v) and were then perfused transcardially with 0.1 M phosphate-buffered saline (pH 7.4), followed by 4% paraformaldehyde in 0.1 M phosphate buffer (PB, pH 7.4). The maxillary tissue with induced periodontitis was excised and immediately immersed in 10% paraformaldehyde in 0.1 M PB (pH 7.4) to avoid dehydration or shrinkage caused by air exposure.

The maxillary tissue was then rinsed with a decalcification solution consisting of 24.4% formic acid and 0.5 N sodium hydroxide for 48 hours. The decalcified tissue was fixed in 10% neutral-buffered formalin for 1 day and then embedded in paraffin using an automated tissue processor, the Shandon Citadel 2000 (Thermo Scientific, Waltham, Massachusetts, United States), and the Shandon Histostar embedding center (Thermo Scientific). From each paraffin block, 5-μm thick sections were cut using an automatic microtome (RM2255, Leica Biosystems, Nussloch, Germany). The sections were mounted on glass slides and stained with H&E. The stained samples were examined under an electron microscope (Nikon Eclipse 80i) at ×40 magnification in five fields of view.

## Results

### Phytochemical Screening

The phytochemical screening results of extracts of *P. pellucida* show the extracts contain all the tested compounds except for saponins, triterpenoids, and steroids, as shown in Table 1 .

**Table 1:** Phytochemical screening of extract of *Peperomia pellucida* (L.) Kunth

Compound class	Result
Alkaloid	+
Polyphenol	+
Tannin	+
Flavonoid	+
Quinone	+
Saponin	−
Monoterpenoids and sesquiterpenoids	−
Steroid and triterpenoids	+

### Antibacterial Activity

**Fig. 3:**
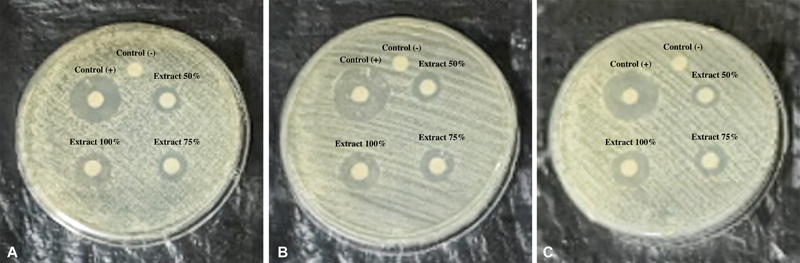
The results of the inhibitory test conducted using maceration and reflux extracts. Panels ( **A** – **C** ) display the inhibition zones at concentrations of 25, 50, 75, and 100%, alongside positive and negative controls, against *Porphyromonas gingivalis* .

The antibacterial activity of pepper elder leaf ( *P. pellucida* (L.) Kunth) extracts against *P. gingivalis* was evaluated using the disc diffusion method, indicated by clear zones around the paper discs, as shown in Fig. 1 . The diameter of the clear zones was measured using a caliper. Each concentration (25, 50, 75, and 100%) was tested three times, as illustrated in Fig. 3 .

Table 2 presents the antibacterial activity of *P. pellucida* extract at varying concentrations, aquades, and CHX against *P. gingivalis* , assessed through the zone of inhibition (mm). The aquades group showed no antibacterial effect, with a mean inhibition zone of 0 mm, categorized as “No Response.” On the other hand, the CHX group displayed the highest antibacterial activity, with a mean inhibition zone of 20.68 ± 0.584 mm, classified as “Very Strong.” The *P. pellucida* extract exhibited a concentration-dependent increase in antibacterial activity. At 25%, the extract showed a mean inhibition zone of 10.18 ± 0.029 mm, which increased to 12.20 ± 0.150 mm at 50%, 13.78 ± 0.176 mm at 75%, and 15.52 ± 0.104 mm at 100%, all categorized as “Strong.” These results highlight that *P. pellucida* extract has strong antibacterial activity across all tested concentrations, with its efficacy improving as the concentration increases, although it remains slightly less effective compared with CHX, which demonstrated very strong antibacterial activity.

**Table 2:** Inhibitory values of pepper elder leaf maceration and reflux extracts

Groups	Zone of inhibition (mm)	Mean ± SD	Interpretation
Aquades	0	0	
	0		No response
	0		
CHX	20.05	20.68 ± 0.584	
	20.80		Very strong
	21.20		
*Peperomia pellucida* extract 25%	10.20	10.18 ± 0.029	Strong
	10.15		
	10.20		
*Peperomia pellucida* extract 50%	12.05	12.20 ± 0.150	Strong
	12.20		
	12.35		
*Peperomia pellucida* extract 75%	13.80	13.78 ± 0.176	Strong
	13.95		
	13.60		
*Peperomia pellucida* extract 100%	15.55	15.52 ± 0.104	Strong
	15.40		
	15.60		

Abbreviations: CHX, chlorhexidine-treated; SD, standard deviation.

### Assessment of PBI and Gingival Index

The following shows the PBI scores for rats with induced periodontitis in the untreated group, the group treated with *P. pellucida* extract, and the group treated with CHX 0.2%. According to Table 3 , on day 0, the negative control group has the highest average PBI of 3.33 and the baseline group has the lowest at 0. On day 3, the negative control group still has the highest average PBI of 3.33, while the baseline group remains the lowest at 0. On day 5, the negative control group shows the highest average PBI of 3.17, with the baseline group having the lowest at 0. On day 7, the positive control group exhibits the highest average PBI of 2.33, and the baseline group has the lowest at 0. For the results of the GI measurement based on Table 3 , the group receiving the *P. pellucida* leaf extract showed mean GI values of 2.800, 1.400, 1.300, and 0.600 on days 0, 3, 5, and 7, respectively. The administration of *P. pellucida* leaf extract resulted in a decrease in the GI from day 0 to 7 ( Table 4 ).

**Table 3:** Papillary Bleeding Index (PBI) and gingival index (GI) of induced periodontitis rats by group

Groups	PBI			GI		
	Mean	Standard deviation	Min–Max	Mean	Standard deviation	Min–Max
Day 0						
No periodontitis	0.000	0.000	0.0–0.0	0.000	0.000	0.0–0.0
No treated	3.330	0.820	2.0–4.0	2.833	0.408	2.0–3.0
CHX-treated	3.000	1.220	1.0–4.0	3.000	0.000	3.0–3.0
Extract-treated	3.200	0.840	2.0–4.0	2.800	0.447	2.0–3.0
Day 3						
No periodontitis	0.000	0.000	0.0–0.0	0.000	0.000	0.0–0.0
No treated	3.333	0.820	2.0–4.0	2.333	0.258	2.0–2.5
CHX-treated	2.600	0.890	2.0–4.0	1.900	0.224	1.5–2.0
Extract-treated	1.600	0.890	1.0–3.0	1.400	0.224	1.0–1.5
Day 5						
No periodontitis	0.000	0.000	0.0–0.0	0.000	0.000	0.0–0.0
No treated	3.17	0.750	2.0–4.0	1.917	0.204	1.5–2.0
CHX-treated	1.800	1.480	0.0–4.0	1.600	0.418	1.0–2.0
Extract-treated	1.000	0.710	0.0–2.0	1.300	0.274	1.0–1.5
Day 7						
No periodontitis	0.000	0.000	0.0–0.0	0.000	0.000	0.0–0.0
No treated	2.330	1.030	1.0–4.0	1.833	0.258	1.5–2.0
CHX-treated	1.600	0.890	1.0–3.0	1.200	0.274	1.0–1.5
Extract-treated	1.200	1.100	0.0–3.0	0.600	0.548	0.0–1.5

Abbreviation: CHX, chlorhexidine-treated.

**Table 4:** Paired difference test between groups

Day	Extract-treated vs. no periodontitis		Extract-treated vs. no treated		Extract-treated vs. CHX-treated	
	*p* -Value					
	PBI	GI	PBI	GI	PBI	GI
Day 0	** 0.004 a**	** 0.004 a**	0.836	0.931	0.940	0.690
Day 3	0.069	** 0.004 a**	0.059	** 0.004 a**	0.307	** 0.032 a**
Day 5	0.141	** 0.004 a**	0.155	** 0.009 a**	0.466	0.310
Day 7	0.051	** 0.030 a**	0.401	** 0.009 a**	0.528	0.095

Abbreviations: CHX, chlorhexidine-treated; GI, gingival index; PBI, Papillary Bleeding Index.

a Original data are tested using the Mann-Whitney test. Statistical significance is determined by a p-value of < 0.05 and are set in bold.

### Histological Assessment

A total of 24 rats were used in this study and divided into four groups: no periodontitis group, no treatment group, CHX-treated group, and extract group. Two rats were randomly selected from each group, and tissue samples from the periodontitis-induced area were collected for histological examination. The study focused on analyzing the number of osteoclasts, osteoblasts, fibroblasts, macrophages, and PMNs, which are closely associated with the severity of periodontitis. The stained samples were viewed under electron microscope (Nikon Eclipse 80i) at ×40 magnification in five fields of view.

The normality of numerical data was assessed using the Shapiro–Wilk test ( Table 5 ). For the variable osteoclasts, the CHX-treated group exhibited a *p* -value greater than 0.05 ( *p* > 0.05), indicating a normal data distribution, while the no periodontitis, no treatment, and extract-treated groups had *p* -values less than 0.05 ( *p* < 0.05), suggesting a nonnormal distribution. For the variable osteoblasts, all groups (no periodontitis, no treatment, CHX-treated, and extract-treated) showed *p* -values greater than 0.05 ( *p* > 0.05), indicating normal data distribution. Similarly, for the variable fibroblasts, all groups exhibited *p* -values greater than 0.05 ( *p* > 0.05), confirming normal data distribution. For the variable macrophages, the no treatment group had a *p* -value greater than 0.05 ( *p* > 0.05), indicating normal distribution, whereas the no periodontitis, CHX-treated, and extract-treated groups had *p* -values less than 0.05 ( *p* < 0.05), indicating a nonnormal distribution. Lastly, for the variable PMN, the no periodontitis, no treatment, and extract-treated groups showed *p* -values greater than 0.05 ( *p* > 0.05), indicating normal distribution, while the CHX-treated group displayed a *p* -value less than 0.05 ( *p* < 0.05), suggesting a nonnormal distribution.

**Table 5:** Normality test of numeric data

Variable	Groups	*p* -Value	Data distribution
Osteoclast	No periodontitis	0.0001**	Not normally
	No treatment	0.0001**	Not normally
	CHX-treated	1.000	Normal
	Extract-treated	0.0001**	Not normally
Osteoblast	No periodontitis	0.135	Normal
	No treatment	0.717	Normal
	CHX-treated	0.282	Normal
	Extract-treated	0.471	Normal
Fibroblast	No periodontitis	0.839	Normal
	No treatment	0.565	Normal
	CHX-treated	0.559	Normal
	Extract-treated	0.968	Normal
Macrophage	No periodontitis	0.008*	Not normally
	No treatment	0.245	Normal
	CHX-treated	0.007*	Not normally
	Extract-treated	0.017*	Not normally
PMN	No periodontitis	0.361	Normal
	No treatment	0.099	Normal
	CHX-treated	0.007*	Not normally
	Extract-treated	0.426	Normal

Abbreviations: CHX, chlorhexidine-treated; PMN, polymorphonuclear neutrophil.

Note: The *p* -value was calculated using the Shapiro–Wilk test. A *p* -value greater than 0.05 ( *p* > 0.05) indicates that the data are normally distributed, while a *p* -value less than 0.05 ( *p* < 0.05) indicates that the data are not normally distributed. Statistical significance is determined at *p* < 0.05.

The homogeneity of numerical data was assessed using the Levene test. The results showed that for the variables osteoclasts, osteoblasts, fibroblasts, and macrophages, the *p* -values were less than 0.05 ( *p* < 0.05), indicating nonhomogeneous data. Conversely, for the variable PMNs, the *p* -value was greater than 0.05 ( *p* > 0.05), indicating homogeneous data ( Table 6 ).

**Table 6:** The homogeneity test

Variable	*p* -Value	Data distribution
Osteoclast	0.002*	Nonhomogeneous
Osteoblast	0.031*	Nonhomogeneous
Fibroblast	0.0001*	Nonhomogeneous
Macrophage	0.002*	Nonhomogeneous
PMN	0.105	Homogeneous

Abbreviation: PMN, polymorphonuclear neutrophil.

Note: The *p* -value was calculated using the Levene test. A *p* -value greater than 0.05 ( *p* > 0.05) indicates homogeneous data, while a *p* -value less than 0.05 ( *p* < 0.05) indicates nonhomogeneous data. Statistical significance was determined at *p* < 0.05.

Table 7 presents the mean values of the number of osteoclasts, osteoblasts, fibroblasts, macrophages, and PMN cells for each group. The *p* -values for each variable are < 0.05, indicating statistically significant differences in the mean values across the four groups.

**Table 7:** Comparison of histological examination results among the four groups

Groups	Osteoclasts		Osteoblasts		Fibroblasts		Macrophages		PMN	
	Mean ± SD	*p* -Value	Mean ± SD	*p* -Value	Mean ± SD	*p* -Value	Mean ± SD	*p* -Value	Mean ± SD	*p* -Value
No periodontitis	0.20 ± 0.632	0.023*	10.20 ± 3.853	0.001*	23.50 ± 6.078	0.0001**	2.30 ± 0.823	0.012*	5.10 ± 2.079	0.005*
No treatment	0.40 ± 0.699		16.80 ± 5.245		59.30 ± 17.263		3.60 ± 0.966		5.10 ± 2.183	
CHX-treated	0.00		7.30 ± 2.163		5.40 ± 3.340		1.60 ± 1.955		2.40 ± 1.075	
Extract-treated	0.60 ± 0.516		11.40 ± 3.806		28.70 ± 8.680		2.10 ± 0.876		4.50 ± 1.841	

Abbreviations: ANOVA, analysis of variance; CHX, chlorhexidine-treated; PMN, polymorphonuclear neutrophil; SD, standard deviation.

Note: For numerical data, the *p* -value is tested using one-way ANOVA when the data are normally distributed and have homogeneous variances. If the data are not normally distributed or the variances are not homogeneous, the Kruskal–Wallis test is used as an alternative. Statistical significance is indicated by a *p* -value of < 0.05. An asterisk (*) denotes a *p* -value of < 0.05, indicating statistical significance. A single asterisk (*) signifies significance at < 0.05, and a double asterisk (**) signifies significance at < 0.01.

The results of the comparative analysis of the four groups in Table 7 indicate statistical significance; thus, after the Kruskal–Wallis test, a post hoc analysis using the Mann–Whitney test was conducted to explain the comparisons between each variable among the groups, with the results presented in Table 8 . The number of osteoclasts showed no significant difference in the extract group compared with both the group without induced periodontitis and the group with induced periodontitis, with *p* -values of 0.105 and 0.393, respectively. A similar result was observed for the PMN variable, where the comparisons between the extract group and both the group without induced periodontitis and the group with induced periodontitis showed no significant differences, with *p* -values of 0.529 and 0.631, respectively.

**Table 8:** Comparison between two groups of each histological examination variable

Variable	Groups		*p* -Value
Osteoclast	No treatment	No periodontitis	0.529
	CHX-treated	No periodontitis	0.739
	CHX-treated	No treatment	0.280
	Extract-treated	No periodontitis	0.105
	Extract-treated	No treatment	0.393
	Extract-treated	CHX treatment	0.023*
Osteoblast	No treatment	No periodontitis	0.007*
	CHX-treated	No periodontitis	0.075
	CHX-treated	No treatment	0.0001*
	Extract-treated	No periodontitis	0.579
	Extract-treated	No treatment	0.023*
	Extract-treated	CHX treatment	0.015*
Fibroblast	No treatment	No periodontitis	0.0001**
	CHX-treated	No periodontitis	0.0001**
	CHX-treated	No treatment	0.0001**
	Extract-treated	No Periodontitis	0.143
	Extract-treated	No Treatment	0.0001**
	Extract-treated	CHX treatment	0.0001**
Macrophage	No treatment	No periodontitis	0.009*
	CHX-treated	No periodontitis	0.218
	CHX-treated	No treatment	0.035*
	Extract-treated	No periodontitis	0.631
	Extract-treated	No treatment	0.004*
	Extract-treated	CHX treatment	0.247
PMN	No treatment	No periodontitis	1.000
	CHX-treated	No periodontitis	0.003*
	CHX-treated	No treatment	0.001*
	Extract-treated	No periodontitis	0.529
	Extract-treated	No treatment	0.631
	Extract-treated	CHX treatment	0.011*

Abbreviations: CHX, chlorhexidine-treated; LSD, least significant difference; PMN, polymorphonuclear neutrophil.

Note: For numerical data, the *p* -value is tested using the LSD test if the data are normally distributed. If the data are not normally distributed or have nonhomogeneous variances, the Mann–Whitney test is used as an alternative. Ordinal data are tested using the Mann–Whitney test. Statistical significance is determined by a *p* -value of < 0.05. An asterisk (*) indicates a *p* -value of < 0.05, denoting statistical significance. A single asterisk (*) signifies significance at < 0.05, and a double asterisk (**) signifies significance at < 0.01.

In histological observations, the number of osteoblasts, fibroblasts, and macrophages did not show significant differences between the periodontitis-induced group treated with *P. pellucida* extract and the group without induced periodontitis, with *p* -values of 0.579, 0.143, and 0.631 for osteoblasts, fibroblasts, and macrophages, respectively. However, comparisons between the extract group and the periodontitis-induced group without treatment showed significant differences, with *p* -values of 0.023, 0.0001, and 0.004, respectively.

**Fig. 4:**
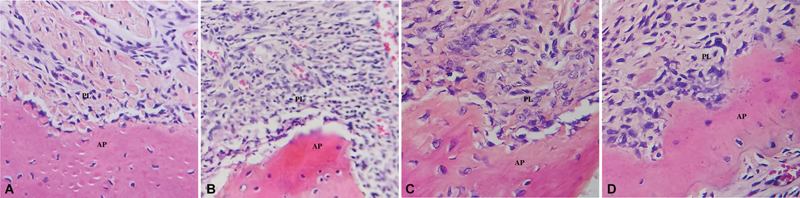
Histological profile of periodontal tissue in rats induced with periodontitis. The image shows inflammatory cell infiltration, collagen degradation, and alveolar bone loss, which are characteristic features of periodontitis. Hematoxylin and eosin staining, magnification 40 × . ( **A** ) No Periodontitis, ( **B** ) No treatment periodontitis induced, ( **C** ) CHX-treated periodontitis induced, ( **D** ) *P.pellucida* extract-treated periodontitis induced.

Fig. 4 shows the histological images of the anterior region of the maxilla tissue in rats, the areas induced with periodontitis for the no treatment, CHX-treated, and extract-treated groups. In the group without periodontitis induction, numerous fibroblasts are present, while osteoblasts are observed in smaller numbers within the bone tissue. In the no treatment periodontitis-induced group, there is significant infiltration of acute and chronic inflammatory cells, alongside increased vasodilation, indicating acute inflammation. In the CHX-treated group, which involves periodontitis-induced rats, many fibroblasts and macrophages are evident in the periodontal tissue, suggesting a reduction in acute inflammation. In the extract-treated group, a substantial presence of fibroblasts is clearly visible in the histological image.

## Discussion

The phytochemical screening of *P. pellucida* extract revealed the presence of various bioactive compounds, including alkaloids, polyphenols, tannins, flavonoids, quinones, monoterpenoids, and sesquiterpenoids. These findings align with the antibacterial activity observed in this study, demonstrate the potential of *P. pellucida* extract as a natural antibacterial agent against *P. gingivalis* , a key pathogen in periodontitis. The extract exhibited dose-dependent antibacterial activity, with higher concentrations producing larger zones of inhibition.

Alkaloids possess antibacterial properties that can lyse bacterial cell walls by disrupting the peptidoglycan components within the bacterial cells. Flavonoids can act as anti-inflammatory agents by blocking the activity of cyclooxygenase (COX)-1 and COX-2 enzymes, inhibiting prostaglandin production, and preventing the accumulation of leukocytes and neutrophil degranulation. This, in turn, directly reduces the release of arachidonic acid by neutrophils and inhibits histamine release. Under normal conditions, leukocytes can move freely along the endothelial walls. During inflammation, endothelial-derived mediators and complement factors cause leukocyte adhesion to the endothelial walls. The administration of flavonoids can reduce leukocyte count and complement activation, thereby decreasing leukocyte adhesion to the endothelium and ultimately reducing the inflammatory response. The antibacterial mechanism of flavonoids involves damaging the proteins that form bacterial cytoplasm by affecting the permeability of their cell membranes, leading to an ion imbalance within the cell. Tannins can lyse bacteria and cause bacterial death by inhibiting transcription processes, preventing the cells from synthesizing proteins. 50 51 52

The active compounds in *P. pellucida* can aid in the wound healing process through various cellular mechanisms, functioning as anti-inflammatory, antimicrobial, and antioxidant agents. 53 Phenols, as antibacterial agents, act as toxins in the protoplasm, damaging and penetrating cell walls, and precipitating bacterial cell proteins. Additionally, compounds such as alkaloids, flavonoids, and tannins have antimicrobial activity. The antimicrobial mechanism of alkaloids involves inhibiting peptidoglycan components in bacteria, preventing the formation of microbial cell walls, making the cells more prone to lysis. Saponins, tannins, and alkaloids are also believed to inhibit reactive oxygen species. 50 54 55 56

In the process of extract preparation, several factors can influence the content of the extract. These factors include, first, biological factors such as the type of plant, location of the plant, time of harvest, plant age, and the method of plant material storage. Second, chemical factors including the types of compounds and active components present, extraction methods, and solvents used. 13 57 58 59 60

Based on the clinical parameter assessments of PBI and GI, it is evident that the administration of *P. pellucida* extract to rats with experimentally induced periodontitis from day 0 to 7 can help reduce both PBI and GI. This effect is attributed to the presence of several anti-inflammatory and antibacterial chemical compounds in the *P. pellucida* plant, such as alkaloids, flavonoids, and tannins. This finding aligns with the research, which demonstrated that *P. pellucida* extract has anti-inflammatory activity against carrageenan-induced edema in rat paws. 29

Rats and other organisms possess a natural ability to heal from certain conditions through the wound healing process without external assistance. However, if the body's natural ability to repair itself is diminished and wound management is inadequate, the wound healing process can be hindered. 52 61 62 The wound healing process involves several phases, including the coagulation and inflammatory phases. During this stage, cellular and vascular responses occur as a result of tissue injury, with platelets playing a role in reducing inflammatory factors that trigger the accumulation of platelets, leukocytes, and other fibroblasts at the injury site. Neutrophils appear within 24 hours to coagulate the blood, followed by epithelial cells migrating from around the wound toward the edge of the incision in the dermis, forming an epithelial layer that covers the wound. The next phase is cellular proliferation, where cells like fibroblasts begin to develop and produce collagen, a protein crucial for scar tissue formation. These cells help rebuild the damaged tissue structure. The final phase of wound healing is the remodeling phase, aimed at enhancing the newly formed tissue. Scar tissue strength can be increased by reinforcing fibrin fibers within the collagen. Collagenase enzymes play a role in breaking down collagen, transforming gelatinous collagen into more mature collagen. Factors that can influence wound healing in rats involve biological mechanisms similar to the healing process in humans. 52 62

This study aims to evaluate the effects of *P. pellucida* extract on rats experimentally induced with periodontitis in the right and left incisor regions of the maxilla. The measured parameters include the number of osteoblasts, osteoclasts, fibroblasts, macrophages, and PMNs. Osteoblasts play a crucial role in bone formation and periodontal tissue regeneration. Osteoclasts are responsible for bone resorption, a key feature of periodontitis. Fibroblasts are involved in synthesizing collagen and other extracellular matrix components, essential for tissue healing and repair. In this study, there was an increase in fibroblast numbers in the group treated with *P. pellucida* extract, indicating the extract's potential to accelerate periodontal tissue healing by stimulating fibroblasts. Macrophages play a key role in immune response and healing processes through phagocytosis and cytokine production. The study found that no treatment group of rats with periodontitis showed an increased number of macrophages, responding to ongoing inflammation. The administration of *P. pellucida* extract seemed to reduce the number of macrophages, potentially indicating an anti-inflammatory effect of the extract, aiding in controlling excessive immune responses. Overall, the administration of *P. pellucida* extract demonstrated positive effects on various histological parameters associated with inflammation and tissue healing in an experimental rat model of periodontitis. The extract not only reduced inflammation but also supported tissue regeneration by increasing the number of osteoblasts and fibroblasts. 61 62 63 64

Several previous studies similar to ours have explored natural extracts for treating periodontitis. *Curcuma longa* (turmeric) extract, rich in curcumin, has shown anti-inflammatory and antioxidant effects in experimentally induced periodontitis in rats. The administration of *Curcuma longa* extract reportedly reduced inflammatory cell infiltration and alveolar bone resorption, with histological analysis showing decreased osteoclast activity and increased new periodontal tissue formation. 65 66 67 Additionally, green tea extract ( *Camellia sinensis* ), which contains polyphenols like epigallocatechin gallate, has been evaluated in rat models of periodontitis. Histological results indicate that green tea extract can reduce gingival inflammation and bone resorption by inhibiting osteoclast activity and enhancing osteoblast activity. 68 Pomegranate extract has also been studied for its potential in treating periodontitis. In periodontitis-induced rat models, this extract was found to reduce tissue inflammation and alveolar bone damage, with histology showing fewer inflammatory cells and less bone resorption in the pomegranate extract group compared with the control group. 69 *Salvadora persica* (miswak), a traditional natural material long used for oral health, has shown in histological studies on rats with periodontitis that its extract can reduce gingival inflammation and alveolar bone resorption. This extract reportedly has antibacterial, anti-inflammatory, and antioxidant effects, all contributing to periodontal tissue improvement. 70 Some studies also suggest that *Nigella sativa* extract can reduce inflammation and tissue damage in periodontitis-induced rats. 71 Histological analysis shows reduced inflammatory cell numbers, increased osteoblast activity, and decreased bone resorption in the group receiving this extract. These findings suggest the potential of *P. pellucida* as a natural therapeutic agent in managing periodontitis, especially in reducing inflammation and supporting tissue regeneration. Further studies are needed to confirm these effects and understand the mechanisms of this extract in more detail. Although the results in rat models are promising, further laboratory and clinical studies are required to ensure the effectiveness and safety of these materials in treating periodontitis. 66

## Conclusion

Based on the findings of this study, *P. pellucida* (L.) Kunth extract contains alkaloids, polyphenols, tannins, flavonoids, quinones, monoterpenoids, and sesquiterpenoids. The extract exhibits antibacterial activity against *P. gingivalis* and demonstrates anti-inflammatory properties, as evidenced by clinical parameters such as the PBI and GI, as well as histological examination showing reduced inflammatory cell infiltration in rats induced with periodontitis.
